# Reliability in One-Repetition Maximum Performance in People with Parkinson's Disease

**DOI:** 10.1155/2012/928736

**Published:** 2011-10-26

**Authors:** Thomas A. Buckley, Christopher J. Hass

**Affiliations:** ^1^Department of Health and Kinesiology, Georgia Southern University, Statesboro, GA 30460, USA; ^2^Department of Applied Physiology and Kinesiology and Movement Disorders Center, University of Florida, Gainesville, FL 32611, USA

## Abstract

Strength training is an effective modality to improve muscular strength and functional performance in people with Parkinson's disease (PWP). One-repetition maximum (1-RM) is the gold standard assessment of strength; however, PWP suffer from day-to-day variations in symptom severity and performance characteristics, potentially adversely affecting the reliability of 1-RM performance. Herein, we assessed the reliability of 1-RM in PWP. Forty-six participants completed two sessions of 1-RM testing of knee extension, knee flexion, chest press, and biceps curl at least 72 hours apart. Significantly differences between testing sessions were identified for knee extension (*P* < 0.001), knee flexion (*P* = 0.042), and biceps curl (*P* = 0.001); however, high reliability (ICC > 0.90) was also identified between sessions. Interestingly, almost third of subjects failed to perform better on the second testing session. These findings suggest that 1-RM testing can be safely performed in PWP and that disease-related daily variability may influence 1-RM performance.

## 1. Introduction

Parkinson's disease (PD), a progressive neurological disease which is believed to affect over 1.5 million Americans, results from the degeneration of the dopaminergic neurons in the midbrain and the resulting reduced dopamine availability to the basal ganglia [1, 2]. The cardinal features of PD include rigidity, tremor, bradykinesia, and impaired postural control, and these symptoms are often unpredictable and their severity can fluctuate daily, often termed “day-to-day variability” [3–5]. Further, muscular weakness, identified by Dr. Parkinson as an early symptom of the disease, is also frequently reported by people with Parkinson's (PWP) [6, 7]. However, inconsistent findings in the literature have obscured the elucidation of the underlying mechanism of the apparent weakness, thus, raising the debate if muscular weakness is intrinsic to the disease or a secondary consequence [8, 9]. Muscular weakness, when present in PWP, presents bilaterally and tends to increase as the velocity of movement increases [9]. While the specific contributory neurophysiological mechanisms remain uncertain, bradykinesia, the inability to energize the appropriate muscles to generate forces at a sufficient rate, is thought to be a major contributing factor [8, 10]. Bradykinesia likely results from basal ganglia pathophysiology leading to impairments in both motor programming and execution [11]. Muscular weakness and bradykinesia impair power production, particularly at lighter loads [8]. These reductions in muscular strength and power have been associated with both reduced functional ambulation and impaired dynamic postural stability in PWP [12–14]. As a result many patients with PD receive physical therapy services to counteract these deficits.

Recent reviews have suggested that strength training may be an effective modality to improve strength and functional performance for PWP [15, 16]. Strength training has frequently been combined with other rehabilitative protocols including cueing strategies, aerobic or cardiovascular training, balance training, stretching exercises, and creatine supplementation in the development of global rehabilitation programs [17–25]. These programs have led to increased muscular strength [17–20], reduced bradykinesia [21], and improved cognitive functioning [22, 23]. Further, these improvements have transferred to overall increased quality of life [21, 25] and improved functional performance including gait [26], sit to stand [27, 28], sit to walk [29], and overall functional mobility [18]. It is not surprising, therefore, that strength training programs have become more integrated into successful Parkinson rehabilitation programs.

An important first step in initiating a rehabilitation program is the assessment of baseline function by which therapy-based improvements can be judged. When resistance training is a component of the therapeutic protocol, assessment of baseline strength is paramount. Though multiple options exist, including more subjective manual muscle testing, the accepted gold standard of maximal muscle testing is the use of the one-repetition maximum (1-RM) test [30]. The 1-RM is defined as the maximal weight that can be lifted once with correct lifting technique and is generally considered to have good to excellent (ICC > 0.95) reliability in healthy adults [31, 32]. However, therapists and rehabilitation specialists need to be aware of the determinants of 1-RM testing which include both previous weight training experience and familiarization with the test [33–35]. Further challenging the assessment of muscular performance are disease-specific complications including the prevalent motor fluctuations, random changes in symptoms severity, and noted “on/off” daily variability [36–38]. 

Previous rehabilitation studies in PWP have utilized either one or two sessions of various strength testing protocols to identify the individual's current strength; however, the reliability of these protocols, specifically maximal strength assessment, has not been assessed in this population [17, 20, 24, 26]. Therefore, the purpose of this study was to investigate the reliability of 1-RM testing in mild-to-moderate PWP across two testing sessions. We hypothesized that 1-RM testing would be generally reliable; however, the disease related day-to-day variability associated with PD would result in individuals differences during the testing.

## 2. Methods

### 2.1. Subjects

A total of 46 participants diagnosed with idiopathic PD by a movement disorder neurologist participated in this study (Table 1). Inclusion criteria included a modified Hoehn and Yahr stage 1–3, the ability to ambulate without assistance, and stable response to anti-Parkinson medications. Exclusion criteria included cardiovascular, musculosketal, vestibular disorders, or other neurological conditions beyond PD or recent enrollment in an exercise training program. All participants were tested while clinically “on” approximately 1–1.5 hours following the first medication dose of the day and self-reported that their medicines were working maximally at the time of testing. No participants demonstrated any dyskinesia or freezing during the testing sessions. All participants provided written informed consent prior to participating in the study as approved by the University's Institutional Review Board.

### 2.2. Experimental Procedures

Prior to performing the 1-RM testing sessions, all participants underwent two familiarization sessions, between 48–72 hours apart, to orientate themselves with the exercise equipment. During these sessions the appropriate positioning and lifting techniques were instructed and each subject performed two sets of each exercise at a low-to-moderate resistance level. The following week, the 1-RM tests were performed using cable-loaded resistance machines for knee extension (KE), knee flexion (KF) (New York Barbell, Elmira, NY.), chest press (CP), and biceps curl (BC) (Nautilus Corp, Vancouver, WA.). Both the 1-RM testing protocol and the participants body alignment for each tested closely adhered to the recommendations of the National Strength and Conditioning Association [30]. For each exercise, subjects warmed up with a low resistance and performed 10 repetitions. Thereafter, resistance was increased in incremental loads until failure occurred despite verbal encouragement to continue [17]. In order to be classified as a successful attempt, the subject had to move the weight through the complete range of motion in a controlled manner without compensatory movements (e.g., shifting body position). The 1-RM was determined within 5 attempts for all subjects. 

In order to reduce the potential confounding effects of fatigue, no individual performed more than two 1-RM tests in a given day and at least 72 hours rest was provided between tests. Specifically, on a given test day the subject would perform one upper body and one lower body assessment. All 46 subjects performed the KE 1-RM tests, followed by 25 subjects performing the BC, 24 subjects performing the CP, and 21 subjects performing the KF.

### 2.3. Statistical Analysis

The same investigator tested the participants on both days. A paired sample *T*-test was performed to compare differences between 1-RM during session 1 and session 2 for each of the four exercises. The mean difference and 95% confidence intervals between the two tests were calculated as session 2 minus session 1, such that a positive number indicates an increase in 1-RM during session 2. A frequency distribution was performed for each exercise to identify which test session most commonly represented the higher value. The intraclass correlation coefficient (ICC) was calculated for each exercise with a two-way random effects analysis of variance. Finally, the standard error of the measurement (SEM) was calculated as SEM = SD_baseline_∗√(1 − *r*_test−retest_) [39].

## 3. Results

All subjects completed all 1-RM tests without incident. The paired analysis revealed statistically significant differences in 1-RM performance between the two testing sessions for knee extension, knee flexion, and biceps curl, but not for chest press (Table 2). The intraclass correlation coefficient ranged from 0.91 to 0.97 (Table 2).

Across the four exercises, a total of 116 tests were performed; of these, 11.2% (13 of 116) had identical scores between the two testing sessions. Further, 19.8% (23 of 116) of the evaluations had higher 1-RM values, a mean of 4.6 kg across all 4 exercises, on the first test. Finally, the range of differences between the two testing sessions was 82% of the combined means (54 kg) with one participant increasing their 1-RM by 41% (27 kg) and another subject exhibiting a 41% (27 kg) reduction in 1-RM, both occurred during knee extension exercises, and over half of all participants (51%) had changes of at least 5 kg between test sessions.

## 4. Discussion

Effective and reliable assessment of force production is an integral component in the development of an appropriate physical therapy program. Further, in longitudinal studies it is essential to establish an accurate and reliable baseline performance of strength to compare improvements over time. The purpose of this study was to investigate reliability in 1-RM performance amongst PWP. A primary finding of this study was a significant difference in 1-RM strength between the two sessions for knee extension, knee flexion, and biceps curl exercises in individuals with mild-to-moderate PD despite the subjects performing two orientation sessions in the previous week. However, the tests demonstrated high reliability and the between sessions differences did not exceed the standard error of measurement when collapsed across participants. Interestingly, nearly third of subjects did not increase their 1-RM on the second testing session as would be expected in this inexperienced population. In some cases, the improvements we observed (up to 41% improvement) rival or exceed those reported in many longitudinal training studies [17, 18, 20, 21]. This finding suggests that day-to-day performance variability may play a substantial role in 1-RM strength testing for individuals with mild-to-moderate PD. 

Accurate and reliable baseline testing needs to be conducted to correctly prescribe the treatment protocol and elucidate improvements following exercise programs. The results of this study suggest that more than one baseline 1-RM test needs to be performed, although therapists should not assume improved performance with second-day testing. Indeed, over 30% of subjects failed to improve in 1-RM performance on the second testing session and a between-test range of 54 kg was identified during the leg extension exercise. This finding raises two unique concerns to the development and reporting on the effects of strengthening programs for Parkinson's rehabilitation. First, if the initial 1-RM value is low, the exercise prescription based on this value may not be sufficiently challenging to the individual, thus, potentially limiting the effectiveness of the therapy. Secondly, variable performance raises the risk that the true benefit of the intervention may be masked by a single day poor performance in a population known to experience day-to-day performance variability [5, 40, 41]. The results of this study are similar to recent finding of aerobic capacity in PWP [42]. Katzel and colleagues demonstrated generally high test-retest reliability, however a significant between test session, 0.56 mL/mg/min, difference was noted in VO_2_ peak measurements [42]. Further, almost half of the PWP, failed to improve on the second administration of the maximal test (95% CI of −3.5–4.6 mL/mg/min) [42]. Taken together, these findings provide important considerations in the development of rehabilitation programs for individuals with mild-to-moderate PD.

The phenomenon of day-to-day variability in PWP has been well established in the literature [5, 40, 41, 43]. The symptoms of Parkinson's, both physical and psychological, are often unpredictable and fluctuate from day to day resulting in substantial alterations in activities of daily living and social activities [40, 44]. This is a separate phenomenon from motor fluctuations, abrupt and unpredictable responses to levodopa administration [45]. Further, both hourly and daily variations, potentially due to motor fluctuations or day-to-day variations, in gait rhythm (e.g., velocity, step length, and cadence), have been identified in PWP [46]. The participants in this study were all tested at a consistent time following medication dosage, at their self-described best time of day, and while clinically “on”; so only subtle motor fluctuations could have been a contributing factor to their performance.

The use of 1-RM testing has been examined in a wide range of healthy, aging, and diseased populations [32, 35, 47–54]. In healthy young adults (age 18–30) with strength training experience, the reliability of the 1-RM test is generally considered to be very high (ICC > 0.95) [47, 55]. In healthy older adults, individuals with cardiovascular disease, peripheral obstructive arterial disease, and chronic obstructive pulmonary disease, 1-RM testing is a safe and practical assessment and our results suggest 1-RM testing is also safe amongst the PWP population with comparable reliability [35, 52–54]. Interestingly, Schilling et al. [20] recently found no differences in maximal relative strength testing, reported as maximum strength divided by body weight; however, these tests were separated by 8 weeks, as opposed to 72 hours, and the time between tests may have influenced the relation to our results. The results of the current study suggest that PWP can safely and effectively perform 1-RM testing and, while important differences exist between trials, the overall results are generally reliable. 

Generally speaking, the reliability of 1-RM measures may vary depending on the individuals experience with weight training and their familiarity with the specific exercise being tested [32, 33, 47–49, 55]. Although the number of acceptable familiarization sessions has ranged from one to nine, in healthy inexperienced middle-aged to older populations, one to three familiarization sessions are generally considered to be appropriate before assessing maximal strength [32, 34, 35]. Following familiarization with the equipment, most studies on healthy older adults suggest that two to three 1-RM sessions are required as strength values will increase on subsequent trials [33–35]. While the specific mechanism underlying these improvements in 1-RM performance, when present, is not fully understood, it is generally attributed to improved neural efficiency and activation patterns as well as a learning effect represented by improved posture and exercise execution [33, 56]. Appropriate orientation and familiarization to the testing paradigm is likely of particular importance for PWP who are known to reduce overall activity due to social stigmas, loss of confidence in their coordination, and fear of falling [26, 57]. 

The findings of this study are delimited to this specific protocol, and future studies should address this potential limitation by increasing the number of both familiarization and 1-RM testing sessions to help elucidate the learning effects and the influence of day-to-day variability. Further, additional demographic considerations (e.g., UPDRS scores) and traditional performance variables (e.g., timed get-up and go test) should be explored to identify potential relationships. However, exploratory analysis of our data found no relationship between disease severity as measured by Hoehn and Yahr staging, body weight or initial strength, and the change in performance between testing sessions. While day-to-day variability in PWP is unpredictable, exercise intervention studies should consider a Parkinson's specific graded symptom checklist on the days of the pre- and posttesting to attempt to control for the variability. Finally, future studies should expand these findings by identifying potential relationships between alterations in strength and performance of activities of daily living.

The 1-RM test is generally considered to be the gold standard for assessing maximal muscular strength in an individual and the results of this study suggest that, when using cable-loaded resistance machines, PWP can successfully and safely perform these tests [30]. Thus, physical therapy interventions can effectively be established and monitored with 1-RM testing in the PD population. Whereas healthy older adults typically demonstrate subtle improvements in 1-RM performance with repeat administration over several days, the results of this study suggest that individuals with mild-to-moderate PD demonstrate inconsistencies in 1-RM test performance.

## Figures and Tables

**Table 1:** Participant demographics and anthropometric data. Anthropometric data is presented as mean ± standard deviation. Hoehn and Yahr classification is presented as the actual number of subjects and the percentage of the total (percentage does not add to 100% due to rounding).

Participant characteristics	
Age (years)	62.6 ± 4.8
Height (m)	1.72 ± 0.11
Weight (kg)	86.8 ± 13.8
Disease duration (years)	10.9 ± 9.9
Hoehn & Yahr score	2.3 ± 0.6
Hoehn & Yahr 1	2 (4.3%)
Hoehn & Yahr 1.5	7 (15.2%)
Hoehn & Yahr 2	14 (30.4%)
Hoehn & Yahr 2.5	11 (23.9%)
Hoehn & Yahr 3	12 (26.1%)
Unified Parkinson Disease Rating Scale (UPDRS)*	
Total score	38.0 ± 6.1
Motor score	23.8 ± 4.6
ADL score	12.2 ± 2.2

*UPDRS data was only available on 25 of the 46 subjects.

**Table 2:** One-repetition maximum test results. The session 1-RM values are presented as mean ± standard deviation.

Exercise	First session (kg)	Second session (kg)	Mean session difference (kg) (95% CI)	*T*-test results	ICC (95% CI)	SEM
Knee extension	63.7 ± 28.1	67.7 ± 29.7	4.0 (1.9–6.2)	*P* < 0.001	.96 (.93–.97)	5.7 kg
Knee flexion	27.0 ± 12.7	29.4 ± 13.0	2.4 (0.2–4.7)	*P* = 0.042	.91 (.79–.96)	3.8 kg
Biceps curl	43.9 ± 15.6	46.6 ± 17.6	2.7 (1.2–4.1)	*P* = 0.001	.97 (.92–.98)	2.8 kg
Chest press	57.8 ± 20.6	60.1 ± 20.8	2.3 (−0.2–4.7)	*P* = 0.066	.95 (.90–.98)	4.3 kg

ICC: Intraclass Correlation Coefficient. SEM: Standard Error of Measurement which was calculated as: SEM: SD_baseline_∗√(1 − *r*_test–retest_).
